# Relationship between physical frailty, depressive symptoms and cognitive ability among older Indians

**DOI:** 10.1093/geroni/igaf122.2297

**Published:** 2025-12-31

**Authors:** Muhammad Thalil, Manish Kumar, Shobhit Srivastava, Waad Ali

**Affiliations:** The Pennsylvania State University, University Park, Pennsylvania, United States; International Institute for Population Sciences, Mumbai, California, United States; International Institute for Population Sciences, Mumbai, California, United States; Sultan Qaboos University, Muscat, Masqat, Oman

## Abstract

With a rapidly aging population, physical frailty has become a significant public health concern globally. We examined the associations of physical frailty with depressive symptoms and cognitive functioning among older Indian men and women, while also exploring how the frailty-cognition link differs between those with and without depression. We used data from the Longitudinal Aging Study in India (2017-19), with a sample of 14,652 males and 15,899 females aged ≥ 60 years. Frailty was assessed using a modified version of Fried’s frailty phenotype. Linear regression models were used to examine the associations. The prevalence of frailty was higher in older women than that in older men (32.2% vs. 27.4%). Physical frailty was significantly associated with higher levels of depressive symptoms (β = 0.51; 95% CI: 0.39, 0.64), and poor cognitive performance (β= -1.06; 95% CI: -1.37, -0.75). Frail older women performed worse on cognitive tests than did frail older men (β= -2.14; 95% CI: -2.40, -1.87). In addition, non-frail older women had poorer cognitive performance than frail older men (β= -0.77; 95% CI: -1.22, -0.32). Conversely, stratification by depressive status showed that frailty was associated with cognitive ability, with no difference between individuals with and without depression. We found that frail older individuals, particularly women, have significant mental and cognitive deficits compared with their non-frail counterparts. Appropriate policies and programs should be implemented to reinforce the strength of pre-frail and frail older adults, and women in particular, and maintain improved mental health and cognition in older adults.

